# Double Model Following Adaptive Control for a Complex Dynamical Network

**DOI:** 10.3390/e25010115

**Published:** 2023-01-05

**Authors:** Xiaoxiao Li, Yinhe Wang, Shengping Li

**Affiliations:** 1 School of Automation, Guangdong University of Technology, Guangzhou 510006, China; 2 MOE Key Laboratory of Intelligent Manufacturing, Shantou University, Shantou 515063, China

**Keywords:** double model following adaptive control, complex dynamical network, nodes group, links group

## Abstract

This paper formulates and solves a new problem of the double model following adaptive control (MFAC) of nodes and links in a complex dynamical network (CDN). This is different from most existing studies on CDN and MFAC. Inspired by the concept of composite systems, the CDN with dynamic links is regarded as an interconnected system composed of an interconnected node group (NG) and link group (LG). Guided by the above-mentioned new idea of viewing a CDN from the perspective of composite systems, by means of Lyapunov theory and proposed related mathematical preliminaries, a new adaptive control scheme is proposed for NG. In addition, to remove the restriction that the states of links in a CDN are unavailable due to physical constraints, technical restraints, and expensive measurement costs, we synthesize the coupling term in LG with the proposed adaptive control scheme for NG, such that the problem of double MFAC of nodes and links in CDN is solved. Finally, a simulation example is presented to verify the theoretical results.

## 1. Introduction

A complex dynamical network (CDN) can be thought of as the graph-theoretic model consisting of many time-varying nodes and their connection relationships, which can be used to describe many real networks in the real world—for example, social networks [1], biological neural networks [2], transportation networks [3], and cellular and metabolic networks [4]. The CDN has received a continuously growing interest and has often been a hot subject of research, since it can help us reveal and better understand the structures and functions of real networks, and so far, many valuable theoretical results have also been obtained [5,6,7,8,9,10,11,12].

Inspired by the concept of composite systems, the CDN can be regarded as an interconnected system composed of two subsystems (NG and LG) coupled with each other. Therefore, the dynamic behavior of CDN should originate not only from the NG but also from the LG, and the dynamic behavior of nodes (links) is influenced by the dynamics of links (nodes). Guided by the above viewpoint, the existing research on the synchronization [5,6], tracking [7,8], and consensus [9,10] of CDNs can be regarded as dynamic characteristics of nodes with the assistance of dynamic links. The research on the structural balancing of CDNs can be regarded as dynamic characteristics of links with the assistance of dynamic nodes [11,12]. However, it is worth noting that double model following adaptive control (MFAC) problems of nodes and links in CDNs are ignored in the above-mentioned research.

Obviously, MFAC as an important class of control theory. Many important results on MFAC have been obtained for various linear and nonlinear systems, which have been applied to many fields [13,14,15,16,17]. By observing the literature [13,14,15,16,17], it is easy to see that most existing works on MFAC are mainly aimed at a single system and are limited by the model matching conditions. In contrast, discussion on the MFAC of networks is rarely seen. Therefore, in order to overcome the above limitations in the current research works on MFAC, combined with the above understanding and discussion of CDN, the new problem of double MFAC of nodes and links in CDN has emerged. In fact, the research on double MFAC of nodes and links in CDN also has practical applications. For example, for the study of discretized structural systems [18,19,20,21], the finite element analysis method can be used to discretize it into *N* elements. Then, to understand the above discretized structural systems from the perspective of a CDN, each discretized element is considered as a node in the CDN, and the stiffness variation between discretized elements is regarded as the dynamic link in the CDN. Then, we can utilize the reference model to specify the expected displacement velocity and stiffness variation of discretized elements that can ensure the stability of this system under the interference of external forces. At this time, the stability control problem of continuous structural systems can be transformed into double-MFAC problems of nodes and links in a CDN. Multiple robots present a certain physical dynamic posture according to their respective reference robots (reference target), and the communication protocol (dynamic links) between controlled robots also tracks the communication protocol between its reference robots [22]. Therefore, inspired by the above description, this paper investigates double-MFAC problems of nodes and links in CDN, which can be used to fill the above-mentioned insufficiencies of the existing research.

Motivated by above discussions, this paper mainly focuses on double-MFAC problems of nodes and links in CDN. In this work, the CDN is considered to be an interconnected system composed of NG and LG coupled with each other, where the dynamic equations of NG and LG are modeled by matrix differential equations (MDEs). That is shown more simply in form than the vector differential equations, as in the literature [5,6,7,8,9,10,23,24,25]. This may reduce the difficulty of the mathematical derivation because the matrix-straightening operation and Kronecker-product operation are avoided. In addition, consider the restriction that the states of dynamic links in CDN are unavailable due to physical constraints, technical restraints, and expensive measurement costs. To remove this restriction, this paper not only proposes the adaptive control scheme for NG, but also designs the coupling term in LG based on the mutual coupling between nodes and links in CDN, which is different from the method in [5,6,7,8,9,10]. It also guarantees that nodes and links in CDN can asymptotically follow their respective reference targets. That is, the problem of double MFAC of nodes and links in CDN is solved.

In this paper, we study double-MFAC problems of nodes and links in CDN, compared with the most existing works on studying CDN and MFAC. This paper mainly has the following contributions.

- The problems of double MFAC are first formulated and solved for nodes and links in CDN.
- To solve the double-MFAC problem, the dynamic equations of nodes and links are modeled by matrix differential equations (MDEs), which enables us to employ the matrix algebra methods for system analysis.
- Note that the state information of LG is unavailable, and thus the LG cannot be controlled directly. In order to address this issue, an effective coupling mechanism between NG and LG is proposed based on a new adaptive control scheme synthesized for NG.
- The subsequent theoretical derivation and proof process show that with the proposed coupling mechanism between NG and LG, the strictive model matching conditions in MFAC of a single system (e.g., [26,27]) are no longer required in this paper.

The structure of the rest of this paper is as follows. In Section 2, we propose the mathematical model for CDN, which is considered to be formed by the mutual coupling of NG and LG and give relevant mathematical assumptions. In Section 3, we synthesize the adaptive control scheme for NG and design the effective coupling mechanism between NG and LG to ensure double MFAC of nodes and links in CDN is realized. The illustrative simulation example is given in Section 4 to validate the correctness and effectiveness of the proposed control scheme and the coupling mechanism in this paper. We give the conclusions in Section 5.

Notation: $R^{n}$ denotes the n− dimensional Euclidean space. $R^{m\times q}$ represents the set of m×q matrices. $M^{T}$ denotes the transpose of the matrix *M*. tr{*} denotes the trace of ${}^{\prime }{*}^{\prime }$, and ${}^{‘}{*}^{’}$ denotes the Euclid norm of the vector or the matrix ${}^{‘}{*}^{’}$. diag{⋯} represents a diagonal matrix. $O_{m\times q}$ denotes a m×q zero matrix.

## 2. Model Description

Consider a class of uncertain controlled CDNs consisting of *N* nodes and links between *N* nodes. Define the state vector of the *i*th node as $x_{i}={\left[x_{i1},x_{i2},\ldots ,x_{in}\right]}^{T}\in R^{n}$, the time-varying link weights from node *j* to the node *i* is $l_{ji}=l_{ji}\left(t\right)$, the initial link weights from node *j* to node *i* as ${l_{ji}}^{0}$, and the control input of the *i*th node as $u_{i}={\left[u_{i1},u_{i2},\ldots ,u_{in}\right]}^{T}\in R^{n}$, i,j=1,2,…,N. In this paper, we consider *N* nodes as a whole, called the node group (NG), and all links as a link group (LG). Therefore, the uncertain controlled CDN can be regarded as an interconnected system composed of NG and LG coupled with each other, for which the dynamics are described as follows.

(1) $$\left\{\begin{array}{c} \dot{X}=AX+F\left(X,t\right)+H\left(X,t\right)L_{0}\Gamma +H\left(X,t\right)L\Gamma +U\\ \dot{L}=PL+\Theta \left(X\right) \end{array}\right.$$

where $A\in R^{n\times n}$ and $P\in R^{N\times N}$ signify constant matrices, $F\left(X,t\right)\in R^{n\times N}$ represents a continuous nonlinear matrix function, and $H\left(X,t\right)\in R^{n\times N}$ is the inner coupling matrix of NG. $\Gamma =diag\left\{\alpha_{1},\alpha_{2},\ldots ,\alpha_{N}\right\}\in R^{N\times N}$ represents coupling-strength matrix, where $\alpha_{i}$ is the coupling strength of node *i* in the NG; i=1,2,…,N. $\Theta \left(X\right)\in R^{N\times N}$ refers to coupling term, which describes the coupling relationship between nodes and links. $X\in R^{n\times N}$, $U\in R^{n\times N}$, $L_{o}\in R^{N\times N}$, and $L\in R^{N\times N}$ express the state matrix of NG, the control input matrix of NG, the initial state matrix, and the state matrix of LG, respectively, which are defined as follows. $X\overset{\Delta }{\;=}[x_{1},x_{2},\ldots ,x_{N}]$, $U\overset{\Delta }{\;=}[u_{1},u_{2},\ldots ,u_{N}]$, $L_{0}\overset{\Delta }{\;=}{\left[{l_{ji}}^{0}\right]}_{N\times N}$, $L\overset{\Delta }{\;=}{\left[l_{ji}\right]}_{N\times N}$.

**Remark 1.**  *(i). Equation (1) can be widely used to describe numerous physical and engineering systems in practice [18,28,29,30]. For instance, consider a 1-DOF discretizable structural system with N discretized elements, each of which is regarded a controlled node, and the stiffness variation between discretized elements is regarded as the time-varying link weights. Guided by this idea, the dynamics of the ith discretized element are described as the following differential equation by using Newton’s theorem. $m_{i}{\ddot{z}}_{i}=f_{i}\left(t\right)-f_{di}\left({\dot{z}}_{i}\right)-\sum_{j=1}^{N}[{k_{ji}}^{0}+k_{ji}\left(t\right)]b_{j}\left(z_{j},{\dot{z}}_{j},t\right)$, in which $m_{i}\in R$, $f_{di}\left({\dot{z}}_{i}\right)\in R$, and $f_{i}\left(t\right)\in R$ represent the mass, damping force, and external force of the ith discretized element, respectively. ${k_{ji}}^{0}$ denotes the initial stiffness between the jth discretized element and the ith discretized element, $k_{ji}\left(t\right)$ represents the stiffness variation of the ith discretized element caused by the displacement change of the jth discretized element. The dynamic mathematical model of stiffness variation matrix $K={\left[k_{ji}\left(t\right)\right]}_{N\times N}$ is depicted as $\dot{K}=BK+\Phi \left(Z\right)$, $Z=\left[z_{1},z_{2},\ldots ,z_{N}\right]\in R^{1\times N}$, i,j=1,2,…,N. Then, we regard N discretized elements as a whole (NG), and the stiffness variation matrix as the state variable of LG; therefore, its dynamics model can be co-written in the form of Equation (1) with the following transformations: $x_{i}={\dot{z}}_{i}$, $X=\left[{\dot{z}}_{1},{\dot{z}}_{2},\ldots ,{\dot{z}}_{N}\right]\in R^{1\times N}$, A=0, $F\left(X,t\right)=\left[{m_{1}}^{-1}\left(f_{1}\left(t\right)-f_{d1}\left(x_{1}\right)\right),{m_{2}}^{-1}\left(f_{2}\left(t\right)-f_{d2}\left(x_{2}\right)\right),\cdots {m_{N}}^{-1}\left(f_{N}\left(t\right)-f_{dN}\left(x_{N}\right)\right)\right]\in R^{1\times N}$, $L_{0}={\left[{k_{ji}}^{0}\right]}_{N\times N}$, $L=K={\left[k_{ji}\left(t\right)\right]}_{N\times N}$, $P=B\in R^{N\times N}$, $U=O_{1\times N}$, $\Theta \left(X\right)=\Phi \left(Z\right)\in R^{N\times N}$, $\Gamma =diag\left({m_{1}}^{-1},{m_{2}}^{-1},\ldots ,{m_{N}}^{-1}\right)\in R^{N\times N}$ and $H\left(X,t\right)=[-b_{1}\left(\int_{0}^{t}x_{1}\left(t\right)dt+z_{1}\left(0\right),x_{1},t\right),-b_{2}\left(\int_{0}^{t}x_{2}\left(t\right)dt+z_{2}\left(0\right),x_{2},t\right),\ldots ,-b_{N}(\int_{0}^{t}x_{N}\left(t\right)dt+z_{N}\left(0\right),x_{N},$*

*$t)]\in R^{1\times N}$. (ii). The dynamics model in the theoretical results [11,12,23,31] of the research on CDN can also be co-written in the form of Equation (1) through appropriate transformations. Accordingly, it is reasonable and widely applicable to describe the dynamics of CDN by Equation (1).*

**Assumption 1.**  *Consider the CDN (1): (i). The constant matrix A is unknown, and the matrix function H(X,t) is known and bounded. (ii) There exists an unknown non-negative number such that the inequality $∥F(X,t)∥\leq \delta$ is satisfied, which implies the matrix function F(X,t) is unknown but bounded. (iii) The constant matrix P is a Hurwitz matrix.*

**Remark 2.**  *(i). The assumption that matrix P is a Hurwitz matrix is commonly used in existing works on CDN problems (see for example [24,25,31]). (ii). If Assumption 1 is satisfied, we can obtain the following Lyapunov equation.*

(2) $$P^{T}S+SP=-W$$

*where matrices $W\in R^{N\times N}$ and $S\in R^{N\times N}$ represent given the positive definite symmetric matrix and its corresponding positive definite symmetric matrix solution about Equation (2), respectively. (iii). Many systems, such as a Lorenz chaotic system [23] and Chua circuit chaotic system [23], can satisfy the inequality $∥F(X,t)∥\leq \delta$ in Assumption 1. In addition, an adaptive law can be designed to estimate the unknown bound δ of the nonlinear matrix function F(X,t).*

We propose the reference model for the CDN (1), which is described by the following matrix differential equation.

(3) $$\left\{\begin{array}{c} {\dot{X}}_{m}=A_{m}X_{m}+G\left(X_{m},t\right)L_{m}+B_{m}U_{m}\\ {\dot{L}}_{m}=A_{lm}L_{m}+B_{lm}X_{m} \end{array}\right.$$

where $X_{m}\in R^{n\times N}$ and $L_{m}\in R^{N\times N}$ denote the reference state matrices for NG and LG, respectively. $A_{m}\in R^{n\times n}$, $B_{m}\in R^{n\times m}$, $A_{lm}\in R^{N\times N}$, and $B_{lm}\in {}^{N\times n}$ are constant matrices, $G\left(X_{m},t\right)\in R^{n\times N}$ denotes the continuous nonlinear matrix function, and $U_{m}\in R^{m\times N}$ represents the reference input matrix of NG.

**Remark 3.**  *(i). Since the reference model (3) can also be used to describe the dynamics of CDN, the double-MFAC problem of CDN in this paper means that make the states of the controlled CDN follow the state of the given CDN. More precisely, we aim to make the states of the nodes and links asymptotically follow their respective reference models. It is worth noting that their respective reference models have mutual interconnections. (ii). For the 1-DOF discretized structural system, Equation (3) can be used to describe the formwork in which the structural system maintains stability under axial forces. This means that if displacement velocity of discretized elements and stiffness variation between them can be changed according to Equation (3), the involved structural system can be guaranteed to be stable in the sense shown in references [32,33,34].*

**Assumption 2.**  *The constant matrix $A_{m}$ in Equation (3) is a Hurwitz matrix.*

**Remark 4.**  *(i). Assumption 2 is commonly used in existing works on MFAC problems (see, for example, [17,26,27]). (ii). If Assumption 2 holds, then for any given positive definite symmetric matrix $Q\in R^{n\times n}$, the following Lyapunov equation has the corresponding positive definite symmetric matrix solution $M\in R^{n\times n}$.*

(4) $${A_{m}}^{T}M+MA_{m}=-Q$$

## 3. Main Results

According to Equations (1) and (3), we definite the model following errors for NG and LG in CDN as $E_{X}=X-X_{m}$ and $E_{L}=L-L_{m}$, respectively. Then, in order to achieve double MFAC for NG and LG in CDN, we give the control target as follows.

**The control target.** Consider the CDN (1) composed of the mutual coupling NG and LG, whose reference model is modeled by Equation (3). Suppose that Assumption 1 and Assumption 2 hold, and the state matrices of NG and LG are available and unavailable, respectively. Our objective is to synthesize an adaptive control scheme *U* of NG and design coupling term Θ(X) in LG, such that the CDN (1) asymptotically follows the reference model (3), i.e., $\lim_{t\to +\infty }E_{X}=O_{n\times N}$, $\lim_{t\to +\infty }E_{L}=O_{N\times N}$, in which $O_{n\times N}$ and $O_{N\times N}$ represent the n×N and N×N zero matrices, respectively. Furthermore, the other involved parameters are guaranteed to be bounded.

According to Equations (1) and (3), we can obtain that models following error-dynamic equations for NG and LG are as follows, respectively.

(5) $$\begin{array}{cc} {\dot{E}}_{X}= & \dot{X}-{\dot{X}}_{m}\\ = & AX+F\left(X,t\right)+H\left(X,t\right)L_{0}\Gamma +H\left(X,t\right)L\Gamma +U-A_{m}X_{m}-G\left(X_{m},t\right)L_{m}-B_{m}U_{m}\\ = & A_{m}E_{X}+\left(A-A_{m}\right)X+F\left(X,t\right)+H\left(X,t\right)L_{0}\Gamma +H\left(X,t\right)E_{L}\Gamma +H\left(X,t\right)L_{m}\Gamma \\ \; & +U-G\left(X_{m},t\right)L_{m}-B_{m}U_{m} \end{array}$$

(6) $$\begin{array}{cc} {\dot{E}}_{L}= & \dot{L}-{\dot{L}}_{m}\\ = & PL+\Theta \left(X\right)-A_{lm}L_{m}-B_{lm}X_{m}\\ = & PL-PL_{m}+PL_{m}+\Theta \left(X\right)-A_{lm}L_{m}-B_{lm}X_{m}\\ = & PE_{L}+\left(P-A_{lm}\right)L_{m}+\Theta \left(X\right)-B_{lm}X_{m} \end{array}$$

Based on Assumption 1, we introduce the estimate value $\hat{\delta }$ of unknown bounds δ and the estimate error $\tilde{\delta }=\delta -\hat{\delta }$. Let $K_{p}$ denote the estimate of ${K_{p}}^{*}=A-A_{m}$ and ${\tilde{K}}_{p}={K_{p}}^{*}-K_{p}$ denote its estimate error. In order to achieve the control target proposed in this paper, we synthesized an adaptive control scheme *U* for NG and designed the coupling term Θ(X) in LG, which are shown as follows.

(7) $$U=-K_{p}X-H\left(X,t\right)L_{0}\Gamma -H\left(X,t\right)L_{m}\Gamma +G\left(X_{m},t\right)L_{m}+B_{m}U_{m}+U_{1}$$

(8) $$U_{1}=-\hat{\delta }sign\left(ME_{X}\right)$$

(9) $${\dot{K}}_{p}=\Lambda_{p}ME_{X}X^{T}$$

(10) $$\dot{\hat{\delta }}=\varepsilon ∥{E_{X}}^{T}M∥$$

(11) $$\Theta \left(X\right)=\left(A_{lm}-P\right)L_{m}-S^{-1}H^{T}\left(X,t\right)ME_{X}\Gamma +B_{lm}X_{m}$$

where $sign\left(ME_{X}\right)=\left\{\begin{array}{cc} \frac{ME_{X}}{∥ME_{X}∥}, & E_{X}\neq O_{n\times N}\\ O_{n\times N}, & E_{X}=O_{n\times N} \end{array}\right.$ represents the matrix signal function, ε>0 is an adjustable parameter, and $\Lambda_{p}\in R^{n\times n}$ is the given adjustable positive definite symmetric matrix.

**Remark 5.**  *(i). The adaptive control scheme U for NG is generated by Equations (7)–(10), which includes three parts. The first one $-K_{p}X$ is the feedback term about state matrix X of NG, in which the estimate matrix $K_{p}$ is updated by Equation (9). The second one $-H\left(X,t\right)L_{0}\Gamma -H\left(X,t\right)L_{m}\Gamma +G\left(X_{m},t\right)L_{m}+B_{m}U_{m}$ is the term related to the reference models of NG and LG, in which all the information is known. The last one $-\hat{\delta }sign\left(ME_{X}\right)$ is a robust term to overcome the nonlinear bounded uncertain term F(X,t) involved in NG, in which the unknown bound is estimated by the adaptive law (10). (ii). The adjustable parameters ε in Equation (10) and $\Lambda_{p}$ in Equation (9) are selected while considering a trade-off based on the practical situation.*

By substituting the adaptive control scheme (7)–(10) into the model following error dynamics (Equation (5)), the following formula can be obtained.

(12) $${\dot{E}}_{X}=A_{m}E_{X}+{\tilde{K}}_{p}X+F\left(X,t\right)+H\left(X,t\right)E_{L}\Gamma +U_{1}$$

**Theorem 1.**  *Consider the CDN (1) whose reference model is given by Equation (3). If Assumptions 1 and 2 are satisfied, by employing the synthesized adaptive control scheme (7)–(10) for NG and the designed coupling term (11) in LG, it can be ensured that double MFAC of NG and LG in CDN is realized. That is, $\lim_{t\to +\infty }E_{X}=\lim_{t\to +\infty }\left(X-X_{m}\right)=O_{n\times N}$, $\lim_{t\to +\infty }E_{L}=\lim_{t\to +\infty }\left(L-L_{m}\right)=O_{N\times N}$ hold. $O_{n\times N}$ and $O_{N\times N}$ denote n×N and N×N-dimensional zero matrices, respectively.*

**Remark 6.**  *(1). The detailed proof of Theorem 1 is in Appendix A, please refer to Appendix A at the end of this paper. (2). The steps for applying Theorem 1 are given as follows.*

*Step (i). Propose the reference model (3); determine parameter matrices $A_{m}$, $A_{lm}$, $B_{lm}$, and $B_{m}$; find the nonlinear matrix function $G(X_{m},t)$ and the reference control input matrix $U_{m}$.*

*Step (ii). Determine the coupling strength matrix Γ, the constant matrix P, the inner coupling matrix H(X,t), and the initial state matrices $L_{0}$ and Z(0) in the controlled CDN.*

*Step (iii). Obtain positive definite matrices M and S by solving Lyapunov equations, Equations (4) and (2), respectively. Then, by substituting the above parameters into the designed adaptive control scheme (7)–(10) and the coupling term (11), which ensures the states of NG and LG can asymptotically follow their respective reference models, the double MFAC of NG and LG in CDN is guaranteed.*

## 4. Simulation Example

In this paper—refer to reference [18]—consider axial plane vibration (n=1) of an elastic beam. We used the finite element analysis method to discretize it into *N* elements (N=20), where the stiffness variation was considered as the links between discretized elements. In addition, it should be noted that the elastic restoring force of each discretized element is not only related to its own stiffness, but also related to the stiffness of other discretized elements. Therefore, under the action of axial force, the motion equation of each controlled element with damping force can be expressed as follows.

(13) $$m_{i}{\ddot{z}}_{i}+f_{di}\left({\dot{z}}_{i}\right)+\sum_{j=1}^{N}[{k_{ji}}^{0}+k_{ji}\left(t\right)]z_{j}=f_{i}\left(t\right)+v_{i}$$

where $z_{i}\in R$, $m_{i}\in R$, $f_{di}\left({\dot{z}}_{i}\right)$, and $f_{i}\left(t\right)$ represent the axial displacement, the mass, the damping force, and the axial external force of the *i*th element, respectively. ${k_{ji}}^{0}$ is the initial stiffness (static stiffness) between the *i*th element and the *j*th element. $k_{ji}\left(t\right)$ is the stiffness variation between the *i*th element and the *j*th element, which implies the bending effect of the *j*th element on the *i*th element through the elastic restoring force. $v_{i}\in R$ is the control input of the *i*th element, i,j=1,2,…,N.

The dynamics mathematical model of the stiffness variation for the elastic beam under axial external force is given as follows.

(14) $$\dot{K}=BK+\Phi \left(Z\right)$$

where the stiffness variation matrix $K\in R^{N\times N}$, the constant matrix $B\in R^{N\times N}$, $Z=\left[z_{1},z_{2},\ldots ,z_{N}\right]\in R^{1\times N}$, and the coupling matrix $\Phi \left(Z\right)\in R^{N\times N}$ denotes the coupling relation between displacement velocity and stiffness variation of the discretized elements.

By the transformation $x_{i}={\dot{z}}_{i}$, the motion equations for *N* elements and the dynamics equation of stiffness variation can be rewritten together in the form of Equation (1), in which $X=\left[x_{1},x_{2},\ldots ,x_{N}\right]\in R^{1\times N}$, A=0, $F\left(X,t\right)=\left[{m_{1}}^{-1}\left(f_{1}\left(t\right)-f_{d1}\left(x_{1}\right)\right),{m_{2}}^{-1}\left(f_{2}\left(t\right)-f_{d2}\left(x_{2}\right)\right),\ldots ,{m_{N}}^{-1}\left(f_{N}\left(t\right)-f_{dN}\left(x_{N}\right)\right)\right]\in R^{1\times N}$, $L_{0}={\left[{k_{ji}}^{0}\right]}_{N\times N}$, $L=L\left(t\right)={\left[k_{ji}\left(t\right)\right]}_{N\times N}$, $\Gamma =diag\left[{m_{1}}^{-1},{m_{2}}^{-1},\ldots ,{m_{N}}^{-1}\right]\in R^{N\times N}$, $U=\left[{m_{1}}^{-1}v_{1},{m_{2}}^{-1}v_{2},\ldots .{m_{N}}^{-1}v_{N}\right]\in R^{1\times N}$, $P=B\in R^{N\times N}$, $H\left(X,t\right)=[-(\int_{0}^{t}x_{1}dt+z_{1}\left(0\right)),-(\int_{0}^{t}x_{2}dt+z_{2}\left(0\right)),\ldots ,$$-(\int_{0}^{t}x_{N}dt+z_{N}\left(0\right))]\in R^{1\times N}$, $\Theta \left(X\right)=\Phi \left(Z\right)\in R^{N\times N}$.

Convert the dynamic equations (13) and (14) of the elastic beam into the form of Equation (1) according to the above transformations. Next, based on the form of Equation (1), we give its reference model as shown in Equation (3).

**Remark 7.**  *(i). In this simulation, Equation (3) gives a template that can ensure the stability of the elastic beam in the sense of these studies [32,33,34]. That is to say, as long as the displacement velocity and the stiffness variation of the controlled elastic beam are under the axial external force change according to Equation (3), the stability of the controlled elastic beam in the sense of literature [32,33,34] can be achieved. (ii). From the results in references [35,36], it can be seen that the controller designed in this paper can be regarded as achieving active mass damping (AMD) control.*

Inspired by the stability of structural systems in the sense described in certain papers [18,32,33,34], we determined the parameters involved in Equation (3) in this simulation, which are shown as follows. In this paper, we used the Matlab toolbox for the numerical simulation.

(i). Generate a r×N dimension matrix randomly by using ¯λ=rand(r,N) (r=2); then let $U_{m}={u_{k}}^{m}\cos\left(¯\;\lambda \pi t\right)e^{-2t}$, where ${u_{k}}^{m}>0$ is an arbitrarily, small positive number. The matrix $B_{m}=b_{m}*rand\left(1,r\right)$ with $b_{m}$ is chosen arbitrarily in (0,1) by command “rand(1)", $A_{m}$ is chosen arbitrarily in (−6,0) by command “−6*rand(1)".

(ii). By command “Ω=unifrnd(−1,3,N,N)", generate an N×N dimensional invertible matrix Ω, and ensure that its elements are within the range (−1,3). Then, give a N×N dimension diagonal matrix $\Pi =diag\{{a_{lm}}^{1},{a_{lm}}^{2},\ldots ,{a_{lm}}^{N}\}$, with the negative real numbers ${a_{lm}}^{k}$ chosen arbitrarily by the command “−2*rand(1)", k=1,2,…,N. Then, let $A_{lm}=\Omega^{-1}\Pi \Omega$. Generate the matrix $B_{lm}$ randomly by using the command “rand(N,n)", which can ensure that its elements are selected within the range of (0,1).

In addition, we draw on the experience of literature [18], the parameters and matrices of the controlled elastic beam involved in this simulation are selected according to the following rules.

(a). Given the total length of elastic beam l=20, the length of the *i*th element $l_{i}$, and satisfaction of $\sum_{i=1}^{N}l_{i}=l$, the mass of the *i*th element $m_{i}=\mu_{i}\rho_{i}l_{i}$, in which $\rho_{i}$ is the mass density of the *i*th element chosen arbitrarily by “$\rho_{i}=3*rand\left(1\right)"$; $0<\mu_{i}<1$ is an adjustable parameter. The damp force of the *i*th element was chosen as $f_{di}\left(x_{i}\right)=\varsigma x_{i}$, where ς is a parameter chosen in [1,3]. The external force was chosen as $f_{i}\left(t\right)=a_{i}\sin\left(\omega_{i}\pi t\right)$, for which the amplitude and angular frequency were chosen arbitrarily by “rand(1)" and “5*rand(1)", respectively.

(b). The matrix *B* in Equation (14) is also generated similarly by the above rules of choosing the matrix $A_{lm}$. Obtain the positive definite symmetric matrix *S* by solving the Lyapunov equation, Equation (2), where W=w*eye(N) with w=100. Since we consider the axial plane motion of the elastic beam, *M* is a positive real number in the Lyapunov equation, Equation (4), which can be selected within the appropriate range of (10,20).

(c). The adjustable parameter ε in Equation (10) is selected by ε=2*rand(1). The selection of adjustable parameter $\Lambda_{p}$ is given by the following rules. Firstly, generate an n−order diagonal matrix $\Lambda_{p1}$ randomly according to the command “$\Lambda_{p1}=diag\left(rand\left(n,1\right)\right)"$; then, obtain an n−order orthogonal matrix $\Lambda_{p2}$ by command “$\Lambda_{p2}=orth\left(rand\left(n,n\right)\right)"$; then, choose $\Lambda_{p}={\Lambda_{p2}}^{T}\Lambda_{p1}\Lambda_{p2}$.

(d). The initial state matrices X(0) and L(0) are chosen by randn(1,N) and randn(N,N), respectively.

**Figure 1:**
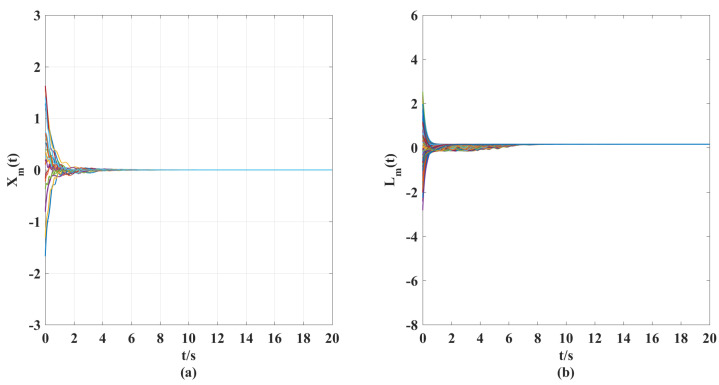
(**a**) Time response curves of reference displacement velocity $X_{m}\left(t\right)$. (**b**) Time response curves of reference stiffness vibration $L_{m}\left(t\right)$.

**Figure 2:**
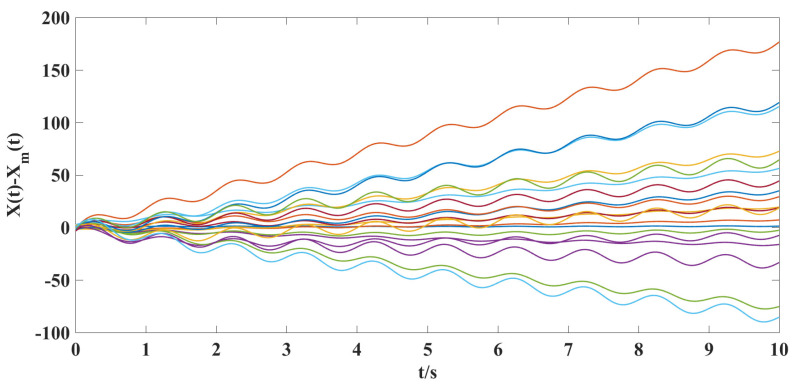
The time response curves of the model following errors for the displacement velocity $X\left(t\right)-X_{m}\left(t\right)$ without a controller.

**Figure 3:**
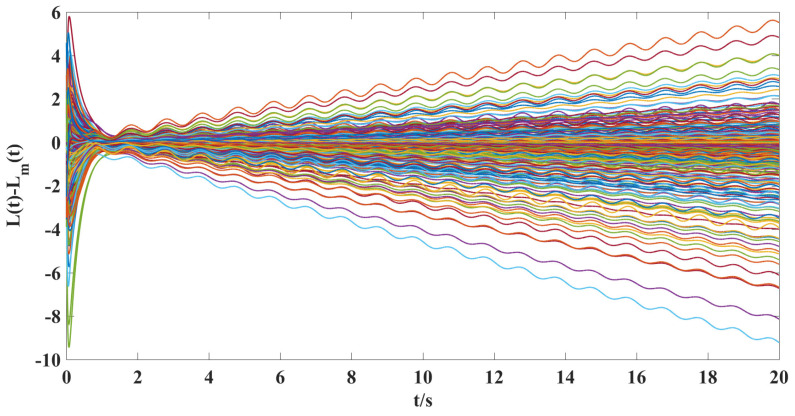
The time response curves of the model following errors for the stiffness vibration $L\left(t\right)-L_{m}\left(t\right)$ without a controller.

**Figure 4:**
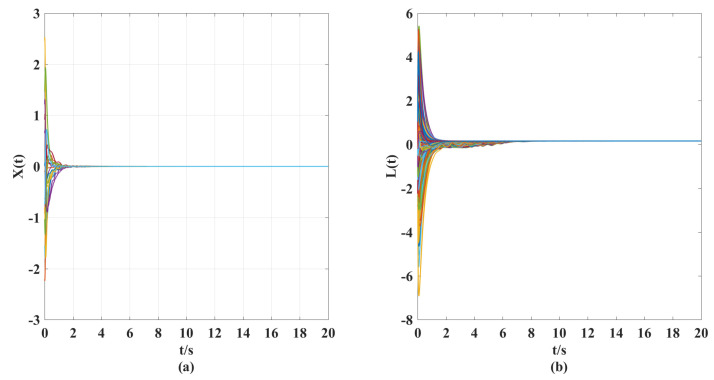
(**a**) Time response curves of displacement velocity X(t) for a controlled elastic beam with a controller and coupling term. (**b**) Time response curves of stiffness vibration L(t) for a controlled elastic beam with a controller and coupling term.

**Figure 5:**
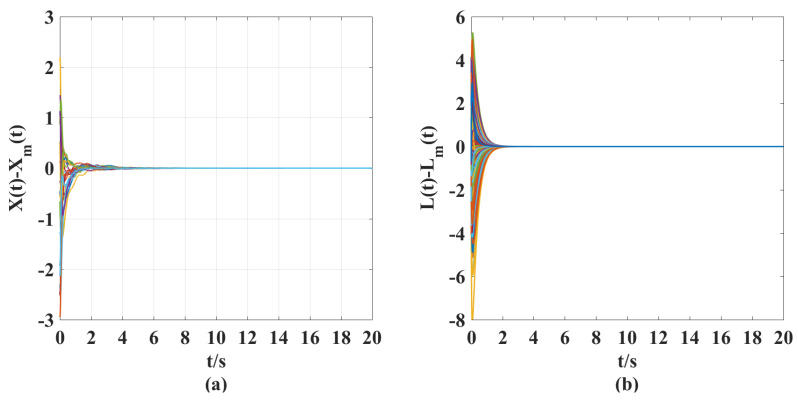
(**a**) Model following error curves of displacement velocity $X\left(t\right)-X_{m}\left(t\right)$ for a controlled elastic beam with a controller and coupling term. (**b**) Model following error curves of stiffness vibration $L\left(t\right)-L_{m}\left(t\right)$ for a controlled elastic beam with a controller and coupling term.

**Figure 6:**
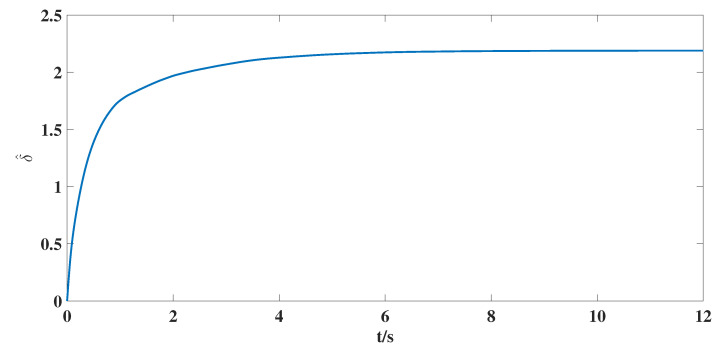
The time response curve of the estimate value $\hat{\delta }$.

**Figure 7:**
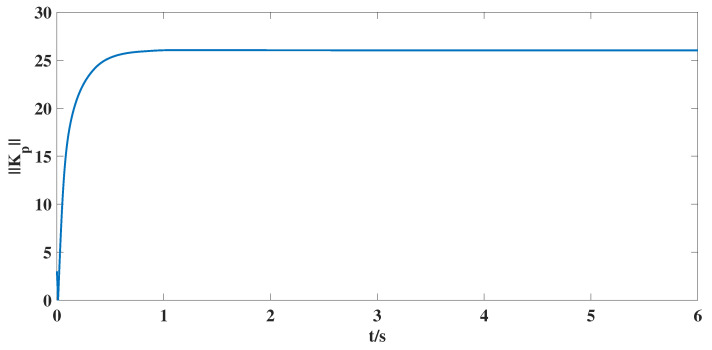
The time response curve for the norm of estimate matrix $K_{p}$ in the controller.

According to the above parameter selection, combined with the synthesized adaptive control scheme for NG, (7)–(10), and the designed coupling term in LG, (11), we can get the following simulation results shown in Figure 1, Figure 2, Figure 3, Figure 4, Figure 5, Figure 6 and Figure 7.

According to the simulation results shown in Figure 1, Figure 2, Figure 3, Figure 4, Figure 5, Figure 6 and Figure 7, we can obtain the following observations.

(i). Figure 1 gives the expected displacement velocity and stiffness variation curves (reference targets) of discretized elements, which can ensure the stability of the elastic beam in the sense of the authors of [32,33,34] under the axial external force.

(ii). From Figure 4 and Figure 5, we can clearly see that under the action of the synthesized adaptive control scheme (Equations (7)–(10)) and designed coupling term (11), the displacement velocity of discretized elements and the stiffness variation between them can asymptotically travel toward their respective reference target values. A comparison of Figure 2, Figure 3, and Figure 5 shows the effectiveness of the control scheme and coupling term designed in this paper.

(iii). Figure 6 shows that the estimate value $\hat{\delta }$ of unknown bound δ for nonlinear matrix function is bounded, and Figure 7 shows that the estimate matrix $K_{p}$ in the control scheme is also bounded, which are required by the control target proposed in this article. At the same time, this also shows the effective estimation for unknown parameters can be guaranteed by designed adaptive laws (9) and (10).

## 5. Conclusions

This paper mainly focused on synthesizing the adaptive control scheme for NG and designing the coupling term in LG to achieve double MFAC of nodes and links in CDN. Firstly, we used matrix differential equations (MDEs) to describe the dynamics characteristics of NG and LG in CDN, respectively, which are different from most existing works on CDN. Then, based on the assumptions proposed in this paper, combined with the Lyapunov stability theorem, the coupling term in LG was designed with the help of the proposed control scheme for NG such that double MFAC of nodes and links in CDN is realized; at the same time, the involved estimation parameters are guaranteed to be bounded. The most outstanding innovation of this paper is to study double-MFAC problems of nodes and links in CDN, while the coupling role between nodes and links is fully considered to remove the restriction on the state information of links. For a dynamics model of links with uncertainties, designing a better adaptive control scheme to make the double MFAC of links and nodes in CDN was implemented, which will be investigated in our future work.

## Data Availability

Not applicable.

## Abbreviations

The following abbreviations are used in this manuscript:

CDN	Complex dynamical network
MFAC	Model following adaptive control
NG	Nodes group
LG	Links group
MDEs	Matrix differential equations

## Appendix A

### Proof of Theorem 1

According to the results in literature [23], we can select the positive definite function $V=V\left(E_{X},E_{L},{\tilde{K}}_{p},\tilde{\delta }\right)=tr\left\{{E_{X}}^{T}ME_{X}\right\}+tr\left\{{E_{L}}^{T}SE_{L}\right\}+tr\left\{{\tilde{K}}_{p}^{T}{\Lambda_{p}}^{-1}{\tilde{K}}_{p}\right\}+\varepsilon^{-1}{\tilde{\delta }}^{2}$ about elements $E_{X}$, $E_{L}$, ${\tilde{K}}_{p}$ and $\tilde{\delta }$, in which positive definite symmetric matrices *M* and *S* are determined by Equations (4) and (2), respectively.

It is well known that the traces of matrices B and C with an appropriate dimension have the following properties.

(a). tr{B+C}=tr{B}+tr{C}

(b). tr{BC}=tr{CB}

(c). $tr\left\{B\right\}=tr\left\{B^{T}\right\}$

(d). $tr\left\{BC\right\}\leq ∥B∥\cdot ∥C∥$

Then, by using Assumptions 1 and 2, the adaptive control scheme (7)–(10) for NG and the coupling term (11) in LG, combined with the above-mentioned properties, we can obtain that the orbit derivative of positive definite function $V=V(E_{X},E_{L},{\tilde{K}}_{p},\tilde{\delta })$ along model following error dynamic Equations (6) and (12) is obtained that.

$$\begin{array}{cc} \dot{V}= & tr\left\{{\dot{E}}_{X}^{T}ME_{X}\right\}+tr\left\{{E_{X}}^{T}M{\dot{E}}_{X}\right\}+tr\left\{{{\dot{E}}_{L}}^{T}SE_{L}\right\}+tr\left\{{E_{L}}^{T}S{\dot{E}}_{L}\right\}+2tr\left\{{{\dot{\tilde{K}}}_{p}}^{T}{\Lambda_{p}}^{-1}{\tilde{K}}_{p}\right\}+2\varepsilon^{-1}\tilde{\delta }\dot{\tilde{\delta }}\\ = & tr\{{\left[A_{m}E_{X}+{\tilde{K}}_{p}X+F\left(X,t\right)+H\left(X,t\right)E_{L}\Gamma +U_{1}\right]}^{T}ME_{X}\}\\ \; & +tr\{{E_{X}}^{T}M\left[A_{m}E_{X}+{\tilde{K}}_{p}X+F\left(X,t\right)+H\left(X,t\right)E_{L}\Gamma +U_{1}\right]\}\\ \; & +tr\left\{{\left[PE_{L}+\left(P-A_{lm}\right)L_{m}+\Theta \left(X\right)-B_{lm}X_{m}\right]}^{T}SE_{L}\right\}+2tr\left\{{{\dot{\tilde{K}}}_{p}}^{T}{\Lambda_{p}}^{-1}{\tilde{K}}_{p}\right\}\\ \; & +tr\left\{{E_{L}}^{T}S\left[PE_{L}+\left(P-A_{lm}\right)L_{m}+\Theta \left(X\right)-B_{lm}X_{m}\right]\right\}+2\varepsilon^{-1}\tilde{\delta }\dot{\tilde{\delta }}\\ = & tr\left\{{E_{X}}^{T}\left[{A_{m}}^{T}M+MA_{m}\right]E_{X}\right\}+tr\left\{{E_{L}}^{T}\left[P^{T}S+SP\right]E_{L}\right\}\\ \; & +2tr\{{E_{X}}^{T}M\left[{\tilde{K}}_{p}X+F\left(X,t\right)+H\left(X,t\right)E_{L}\Gamma +U_{1}\right\}\\ \; & +tr\left\{{E_{L}}^{T}S\left[\left(P-A_{lm}\right)L_{m}+\Theta \left(X\right)-B_{lm}X_{m}\right]\right\}+2tr\left\{{{\dot{\tilde{K}}}_{p}}^{T}{\Lambda_{p}}^{-1}{\tilde{K}}_{p}\right\}+2\varepsilon^{-1}\tilde{\delta }\dot{\tilde{\delta }}\\ = & -tr\left\{{E_{X}}^{T}QE_{X}\right\}-tr\left\{{E_{L}}^{T}WE_{L}\right\}+2tr\left\{{E_{X}}^{T}M{\tilde{K}}_{p}X+{{\dot{\tilde{K}}}_{p}}^{T}{\Lambda_{p}}^{-1}{\tilde{K}}_{p}\right\}\\ \; & +2tr\left\{{E_{X}}^{T}MF\left(X,t\right)-\hat{\delta }{E_{X}}^{T}Msign\left(ME_{X}\right)\right\}+2\varepsilon^{-1}\tilde{\delta }\dot{\tilde{\delta }}\\ \; & +2tr\left\{{E_{X}}^{T}MH\left(X,t\right)E_{L}\Gamma \right\}+2tr\left\{{E_{L}}^{T}S\left[\left(P-A_{lm}\right)L_{m}+\Theta \left(X\right)-B_{lm}X_{m}\right]\right\}\\ \leq & -tr\left\{{E_{X}}^{T}QE_{X}\right\}-tr\left\{{E_{L}}^{T}WE_{L}\right\}+2∥{E_{X}}^{T}M∥\cdot \left[∥F(X,t)∥-\hat{\delta }\right]+2\varepsilon^{-1}\tilde{\delta }\dot{\tilde{\delta }}\\ \; & +2tr\left\{\left[X{E_{X}}^{T}M+{{\dot{\tilde{K}}}_{p}}^{T}{\Lambda_{p}}^{-1}\right]{\tilde{K}}_{p}\right\}+2tr\{{E_{L}}^{T}S[S^{-1}H^{T}\left(X,t\right)ME_{X}\Gamma \\ \; & +\left(P-A_{lm}\right)L_{m}+\Theta \left(X\right)-B_{lm}X_{m}]\} \end{array}$$

(A1) $$\begin{array}{cc} \leq & -tr\left\{{E_{X}}^{T}QE_{X}\right\}-tr\left\{{E_{L}}^{T}WE_{L}\right\}+2\tilde{\delta }\left[∥{E_{X}}^{T}M∥+\varepsilon^{-1}\dot{\tilde{\delta }}\right]\\ = & -tr\left\{{E_{X}}^{T}QE_{X}\right\}-tr\left\{{E_{L}}^{T}WE_{L}\right\}\\ \leq & 0 \end{array}$$

According to inequality (), we can know that the model following errors $E_{X}$ and $E_{L}$ of NG and LG, the estimate errors ${\tilde{K}}_{p}$ and $\tilde{\delta }$ are bounded. Furthermore, by applying the above obtained results to Equations (12) and (6), it can be known that ${\dot{E}}_{X}$ and ${\dot{E}}_{L}$ are also bounded. Then, according to Barbalat Lemma [37,38], we can obtain that $\lim_{t\to +\infty }E_{X}=O_{n\times N}$, $\lim_{t\to +\infty }E_{L}=O_{N\times N}$. Therefore, This completes the proof of Theorem 1.
